# Cardiac tamponade as the initial presentation of systemic lupus erythematosus: a case report and review of the literature

**DOI:** 10.1186/s12969-015-0005-0

**Published:** 2015-03-17

**Authors:** Satish S Maharaj, Simone M Chang

**Affiliations:** Eric Williams Medical Sciences Complex, The University of the West Indies, Champs Fleurs, Trinidad and Tobago

**Keywords:** Systemic lupus erythematosus, Childhood onset, Cardiac tamponade, Pericarditis, Pericardial effusion

## Abstract

Systemic lupus erythematosus (SLE) is an autoimmune disease that can involve any organ system, exhibiting great diversity in presentation. Cardiac tamponade as the initial presentation of childhood onset SLE (cSLE) is rare. We report the case of a 10 year old Afro-Caribbean female who presented with complaints of chest pain, shortness of breath and fever over 4 days. Clinical examination strongly suggested cardiac tamponade which was confirmed by investigations and treated with pericardiocentesis. After a thorough investigation, the underlying diagnosis of SLE was confirmed using the Systemic Lupus International Collaborating Clinics (SLICC) criteria and high dose corticosteroid therapy initiated. A review of recent studies shows that common initial presentations of cSLE include constitutional symptoms, renal disease, musculoskeletal and cutaneous involvement. In presenting this case and reviewing the literature we emphasize the importance of cSLE as a differential diagnosis when presented with pericarditis in the presence or absence of cardiac tamponade. In these patients early diagnosis and treatment is desired and in this regard we also discuss the sensitivity of the SLICC criteria in cSLE.

**Electronic supplementary material:**

The online version of this article (doi:10.1186/s12969-015-0005-0) contains supplementary material, which is available to authorized users.

## Background

Systemic lupus erythematosus (SLE) is an autoimmune disease that can involve any organ system resulting in a great diversity of clinical presentation. Approximately 20% of cases present in childhood. The estimated incidence of childhood onset systemic lupus erythematosus (cSLE) has been reported as 0.28 to 2.22 per 100,000 children and prevalence 6.3 - 9.73 per 100,000 children, with higher frequencies in non-Caucasian populations [1-3]. Pericarditis and pericardial effusions in SLE are well recognized in SLE. Cardiac tamponade is a medical emergency that develops when a pericardial effusion reaches a critical amount, limiting cardiac inflow and leading to hemodynamic compromise. In this case report we present and discuss pericarditis leading to cardiac tamponade as the initial manifestation of cSLE.

## Case presentation

A 10 year old girl of Afro-Caribbean descent presented to the emergency department with complaints of chest pain, shortness of breath and fever over the past 4 days. The left-sided chest pain was described as “squeezing,” and was associated with palpitations. Both the pain and shortness of breath were worse in the supine position and partially relieved on leaning forward. She had no past medical history. However, over the last three months she was becoming increasingly fatigued and noticed significant weight loss, intermittent fevers and arthralgia affecting the wrist and elbow joints. She denied hair loss, oral ulcers, a rash or any medication use. There was no known family history of autoimmune disease.

On examination she had a heart rate of 135 bpm, a respiratory rate of 40 breaths/min, blood pressure of 93/63 mmHg and a temperature of 37.7°C. She was underweight with a body mass index of 13.8 kg/m^2^, had pale conjunctivae and maintained an oxygen saturation of 98% on room air. On cardiovascular examination heart sounds were muffled and the apex beat was diffuse and displaced inferiorly. The pulse was diminished on inspiration. It was difficult to formally assess for pulsus paradoxus due to the patient’s abnormal respiration. Jugular venous distension was evident. Respiratory examination revealed decreased breath sounds at the left lung base which was also dull on percussion. The rest of the examination only proved significant for generalized lymphadenopathy. There was no evidence of joint inflammation, peripheral oedema or clubbing.

**Figure 1:**
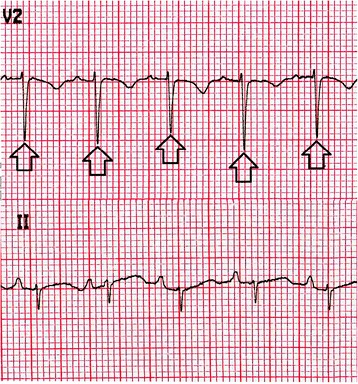
**Electrical alternans was clearly evidenced by the alternating amplitude of the QRS complexes (arrows).**

**Figure 2:**
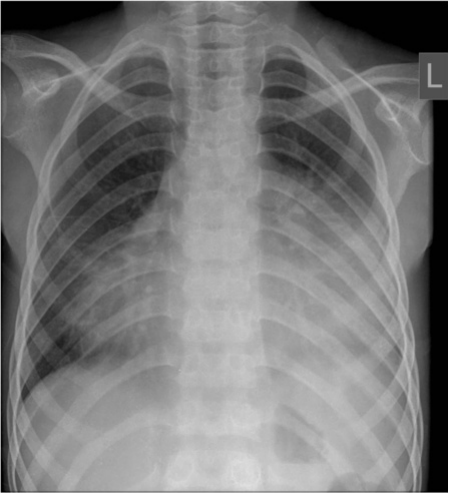
**Posteroanterior chest radiograph at presentation revealed a markedly enlarged cardiac silhouette and a left-sided pulmonary infiltrate.**

**Figure 3:**
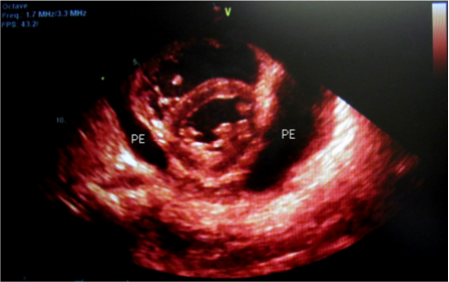
**Two-dimensional transthoracic echocardiography visualized a large circumferential pericardial effusion (PE).**

**Figure 4:**
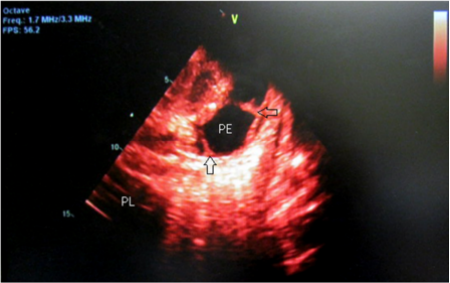
**Two-dimensional transthoracic echocardiography was also remarkable for mobile fibrinous strands (arrows) between the visceral and parietal pericardium, as well as confirmation of a left-sided pleural effusion (PL).**

Electrocardiography showed sinus tachycardia, low voltage and electrical alternans [Figure 1, Additional file 1]. The chest radiograph displayed an enlarged cardiac silhouette with a left-sided pulmonary infiltrate [Figure 2]. Transthoracic echocardiography confirmed features of cardiac tamponade from a large circumferential pericardial effusion, along with mobile intrapericardial fibrinous strands and the pleural effusion [Figures 3 and 4].

Echocardiography guided pericardiocentesis was then performed and 1000 mL of pericardial fluid was drained, immediately bringing relief to the patient. Repeat echocardiography showed a reduced volume of pericardial fluid with no additional reaccumulation and normal heart function.

Laboratory investigations revealed a normocytic anaemia with a normal white cell count and differential. Erythrocyte sedimentation rate (ESR) was 91 mm/hr and C-reactive protein (CRP) was markedly elevated at 191 mg/dL. Cardiac biomarkers, renal and liver function testing were all normal. Urinalysis was within normal limits and showed no proteinuria. HIV testing and a Mantoux tuberculin skin test were negative. The patient’s serum tested strongly positive for antinuclear antibodies (ANA) with a coarse speckled pattern and was also positive for anti-double stranded DNA antibodies (anti-dsDNA). Serum complement levels were low with a C3 of 36 mg/dL (normal range 101 – 186 mg/dL) and C4 of 6.7 mg/dL (normal range 16 – 47 mg/dL). Pericardial fluid cytology showed cells of an inflammatory response with no microorganisms identified on Gram stain or Ziehl-Neelsen stain. There was no evidence of malignancy.

The diagnosis of SLE was established based on the positive clinical and immunologic findings. The patient satisfied 4 of the 17 Systemic Lupus International Collaborating Clinics (SLICC) criteria for classifying SLE, namely serositis, low serum complement levels, positive serum ANA and positive anti-dsDNA antibodies [4]. Supporting the diagnosis was the history of arthralgia, generalized lymphadenopathy and elevated ESR. The patient was started on high dose corticosteroids and discharged soon after with follow up as an outpatient. At the follow up visit she continued to do well and was enrolled in clinic for long term management of cSLE.

## Discussion

SLE is one of the most common autoimmune connective tissue diseases in childhood, where it tends to present more severely than in adults. Despite this, relatively few studies have reported on the clinical features of cSLE at presentation. An understanding of this pattern would help to reduce the frequently reported diagnostic delay in this age group. Therefore, to better understand the spectrum of presenting manifestations of cSLE, we examined six cohorts from different countries that included data on pericarditis [5-10]. While acknowledging the variation in frequencies due to cohort size, patients’ ethnicity and selection biases, certain patterns were clear. The data (summarized in Table 1) showed that common initial presentations of cSLE included constitutional symptoms, renal disease, musculoskeletal and cutaneous involvement. Less frequently involved at cSLE presentation were the neuropsychiatric, pulmonary and cardiac systems, with pericarditis reported in 3–24% of cases at presentation.

**Table 1:** **Frequency of selected presenting clinical features of childhood-onset SLE**

	**Balkaran** ***et al*** [5]	**Spinosa** ***et al.*** [6]	**Abdwani** ***et al.*** [7]	**Hiraki** ***et al.*** [8]	**Agarwal** ***et al.*** [9]	**Gulay** ***et al.*** [10]
**Country**	**Trinidad**	**Brazil**	**Oman**	**Canada**	**India**	**Phillipines**
**Sample size**	**33**	**47**	**50**	**256**	**70**	**78**
*Clinical features (%)*						
Malar rash	36	32	-	61	57	65
Musculoskeletal	70	32	76	61	66	41
Renal disease	64	38	64	45	77	63
Fever	76	34	62	39	94	-
Weight loss	-*	26	52	29	30	-
Ulcers	-	9	-	33	-	54
Alopecia	-	17	36	22	46	40
Pleuritis/ Pleural effusion	9	17	26	12	3	14
Pericarditis/ Pericardial effusion	24	4	22	12	3	15
Neuropsychiatric	-	36	18	16	21	31

* denotes no data was presented for that clinical feature.

Our patient presented with the non specific symptoms of positional chest pain and dyspnea on exertion. Examination revealed tachycardia, tachypnea, hypotension, increased jugular venous pressure and distant heart sounds, leading to the diagnosis of cardiac tamponade. Electrocardiography demonstrated sinus tachycardia, electrical alternans (an alternating QRS amplitude with every other beat) and low voltage, features strongly suggestive of pericardial effusion and tamponade [11,12]. Chest radiography showed an enlarged cardiac silhouette and a left-sided infiltrate, a combination reported to have a strong positive predictive value for pericardial effusion [13]. Echocardiography should be performed if possible, not only because it is the standard to confirm cardiac tamponade [14] but as it can also grade and localize the pericardial effusion, detect pericardial thickening and visualize intrapericardial adhesions. In our patient echocardiography clearly visualized a large circumferential pericardial effusion with characteristic signs of tamponade and also confirmed a left-sided pleural effusion. Within the pericardial effusion, there were partially attached mobile fibrinous strands traversing the visceral and parietal pericardium (Figure 4). This visually dramatic feature has been reported in similar aSLE cases and may be a harbinger of difficult pericardiocentesis [15-17].

Few studies have examined cardiac involvement in cSLE and cardiac tamponade in these patients has not been well-defined. In a 10-year retrospective single center study, Oshiro et al. examined 31 patients (<18 years) diagnosed with cSLE [18]. It was found that 13 patients (42%) had cardiac involvement and 2 patients presented with cardiac tamponade (6%). A more recent multicenter cross-sectional study of 155 cSLE patients (<16 years) reported the initial manifestation of cardiac tamponade in 2 cases (1.3%) [19]. This latter figure is consistent with findings in adult SLE (aSLE) where it is reported 1% of patients present in this way [20]. The much higher incidence reported by Oshiro et al. was likely influenced by the composition of their sample which comprised mainly African-Americans, who are known to have more severe disease. This however raises the issue of whether there is a subset of cSLE patients subject to a higher burden from pericardial disease.

An interesting question is whether cardiac tamponade is a rare occurrence in cSLE, or rather only rare as the initial presentation. Pericarditis can occur at any time during the disease course but appears to be one of the earlier cardiac manifestations. This is clearly seen in a longitudinal study of 256 cSLE patients (<18 years) where out of the 39 patients who had pericarditis at any time (mean follow-up time 3.5 ± 3 years), 30 patients presented at diagnosis [8]. However data on the prevalence of cardiac tamponade throughout the course of cSLE is lacking. In one aSLE series of 395 patients, 10 patients were found to have cardiac tamponade and in 4 patients it was the initial manifestation [20]. Therefore in aSLE it is likely that cardiac tamponade is truly rare, both as the initial manifestation and throughout the disease course. Whether this pattern holds true in cSLE is yet unclear.

We sought to better characterize cardiac tamponade as the initial presentation of cSLE by performing a review of the literature for patients less than 18 years old. Including the present case, 15 cases of cardiac tamponade as the initial manifestation of cSLE were identified using Medline, Scopus, Google Scholar and bibliographies of relevant articles. Of these, 2 cases were excluded from analysis; one due to insufficient data [21] and the other due to possible confounding from growth hormone therapy which was associated with increased risk of immune disease and lupus flares [22]. The 13 remaining cases of cSLE presenting with cardiac tamponade were analyzed and are summarized in Table 2 [17,23-32].

**Table 2:** **Cardiac tamponade as the presenting feature of childhood onset systemic lupus erythematosus**

	**Reference**	**Age (yrs)**	**Sex**	**SLICC criteria**	**Treatment**
*1*	Present case	10	F	Serositis, low serum complement, positive ANA and anti-dsDNA titres.	Pericardiocentesis and oral steroids.
*2*	Sharda [23]	11	F	Serositis, thrombocytopenia, low serum complement, positive ANA and anti-dsDNA titres.	Pericardiocentesis and oral steroids.
*3*	Yiallourides *et al.* [24]	14	F	Hemolytic anemia, serositis, positive ANA and anti-dsDNA titres.	Pericardial drain insertion, oral steroids, intravenous methylprednisolone and two cycles of cyclophosphamide.
*4*	Arabi *et al.* [25]	9	M	Serositis, nephritis, seizures, elevated ANA and anti-dsDNA titres.	Pericardiocentesis with pericardial drain insertion, methylprednisolone pulse therapy followed by oral steroids.
*5*	Arabi *et al.* [25]	11	F	Serositis, elevated anti-dsDNA titres.	Pericardiocentesis, NSAIDs, oral steroids and antimalarial drugs.
*6*	Mohseni *et al.* [26]	14	F	Diagnosed at autopsy; serositis, elevated ANA and anti-dsDNA titres.	Pericardiocentesis.
*7*	Kumar *et al.* [17]	17	F	Serositis, hemolytic anemia, low serum complement, positive antiphospholipid antibody, positive ANA and anti-dsDNA titres.	Aborted pericardiocentesis followed by surgical pericardiectomy, NSAIDs, IV methylprednisolone followed by oral steroid and antimalarial drugs
*8*	Saz *et al.* [27]	3	F	Serositis, positive ANA.	Pericardiocentesis and oral steroid.
*9*	Weich *et al.* [28]	15	F	Serositis, leukopenia, positive anti-dsDNA titre.	Pericardiocentesis with catheter placement, antituberculosis and oral steroid drugs.
*10*	Aiuto *et al.* [29]	14	F	Serositis, nephritis, positive ANA and anti-dsDNA.	Pericardiocentesis and oral steroids.
*11*	Gulati *et al.* [30]	8	F	Serositis, low serum complement, positive ANA and anti-dsDNA.	Pericardiocentesis, antituberculosis treatment and oral steroids.
*12*	Rudra *et al.* [31]	14	F	Serositis, low serum complement, positive ANA and anti-dsDNA.	Pericardiocentesis and oral steroids.
*13*	Lerer [32]	15	F	Serositis, nephritis, positive ANA.	Pericardiocentesis followed by surgical pericardiectomy and oral steroids.

An overwhelming female predominance was noted with only 1 male patient affected. In 5 cases there was a co-existing pleural effusion which was either left-sided or present bilaterally [24,25,27]. Co-existing pleuritis and/or pleural effusion has commonly featured in recent cases of aSLE presenting with tamponade and outside of SLE, bilateral pleural effusions in cardiac tamponade are distinctly rare [33,34]. Hematologic abnormalities were also frequently found including hemolytic anemia, thrombocytopenia and leukopenia [17,24,23,28]. Although not tested for in all cases, 5 patients had low serum complement [17,23,30,31]. This is significant as a low serum complement C4 level was found to be predictive of progression to cardiac tamponade in a series of aSLE patients [35,36]. However, we also note it has been reported that inherited complement deficiencies are a frequent comorbidity in cSLE [19,37]. Therefore the significance of low serum complement in these patients would benefit from further research.

The immediate treatment of cardiac tamponade involves withdrawal of pericardial fluid usually by pericardiocentesis, done in all cases except one which was aborted due to a thickened pericardium with adhesions [17]. When compared to surgical drainage, echocardiography guided pericardiocentesis has been associated with lower morbidity and mortality rates [38]. Some cases required placement of a pericardial drain or less commonly surgical pericardiectomy. Medical management consists of anti-inflammatory medication which typically involves high dose corticosteroids, antimalarials and non-steroidal anti-inflammatory drugs (Table 2). Follow-up is essential to exclude recurrent pericardial effusions and pericardial thickening. In this patient both the erythrocyte sedimentation rate (ESR) and C-reactive protein (CRP) level were elevated. While an elevated ESR is common during a flare, CRP is often normal or mildly elevated. Serositis is one of the few manifestations of SLE that causes both an elevated CRP level and ESR [33,39].

The Systemic Lupus International Collaborating Clinics (SLICC) [4] recently proposed classification criteria addressing perceived weaknesses of the 1997 American College of Rheumatology (ACR) classification criteria [40]. Using the SLICC criteria, our patient was diagnosed with cSLE. Interestingly, the ACR criteria do not include low serum complement C3 and/or C4 levels as a relevant immunologic criterion. As such, our patient was unable to fulfil 4 out of the 11 ACR criteria for classifying SLE. In recent pediatric series, use of the SLICC criteria for the classification of cSLE, showed better sensitivity and led to fewer misclassifications, but was less specific than the ACR criteria [41]. As seen in our patient, low complement levels have been reported as one of the more frequently seen immunologic factors found at presentation in cSLE [41]. The SLICC criteria have also allowed anti-dsDNA antibodies, anti-Sm antibodies and antiphospholipid antibodies to contribute individually to the diagnosis. Atypical presentation is common in cSLE and often leads to major diagnostic delay. By allowing for greater weighting of immunologic criteria the use of the SLICC criteria may be more sensitive for diagnosis in these cases, potentially leading to earlier diagnosis and treatment.

## Conclusion

Cardiac tamponade as the initial presentation of cSLE is rare. More females than males presented in this way, and common co-existing findings included pleuritis and/or pleural effusion, hematologic abnormalities and low serum complement levels. Physicians must consider cSLE in the differential diagnosis of pericarditis and cardiac tamponade and perform appropriate testing for rheumatologic disease.

## Consent

Written informed consent was obtained from the parent of the patient for publication of this Case Report. A copy of the written consent is available for review by the Editor-in-Chief of this journal.

## Additional file

Additional file 1: The 12-lead electrocardiogram on presentation was notable for sinus tachycardia, low voltage (most prominent in limb leads) and electrical alternans (most prominent in precordial leads).

## Abbreviations

ACR: American College of Rheumatology
aSLE: Adult onset systemic lupus erythematosus
ANA: Antinuclear antibody
anti-dsDNA: anti- double stranded DNA
CRP: C-reactive protein
cSLE: Childhood onset systemic lupus erythematosus
ESR: Erythrocyte sedimentation rate
NSAIDs: Nonsteroidal anti-inflammatory drugs
SLICC: Systemic Lupus International Collaborating Clinics
