# Supplementary figures and images for: Cardiac tamponade as the initial presentation of systemic lupus erythematosus: a case report and review of the literature
Source: Pediatr Rheumatol Online J. 2015 Mar 17;13:9. doi: 10.1186/s12969-015-0005-0 (PMC4369869; doi:10.1186/s12969-015-0005-0)

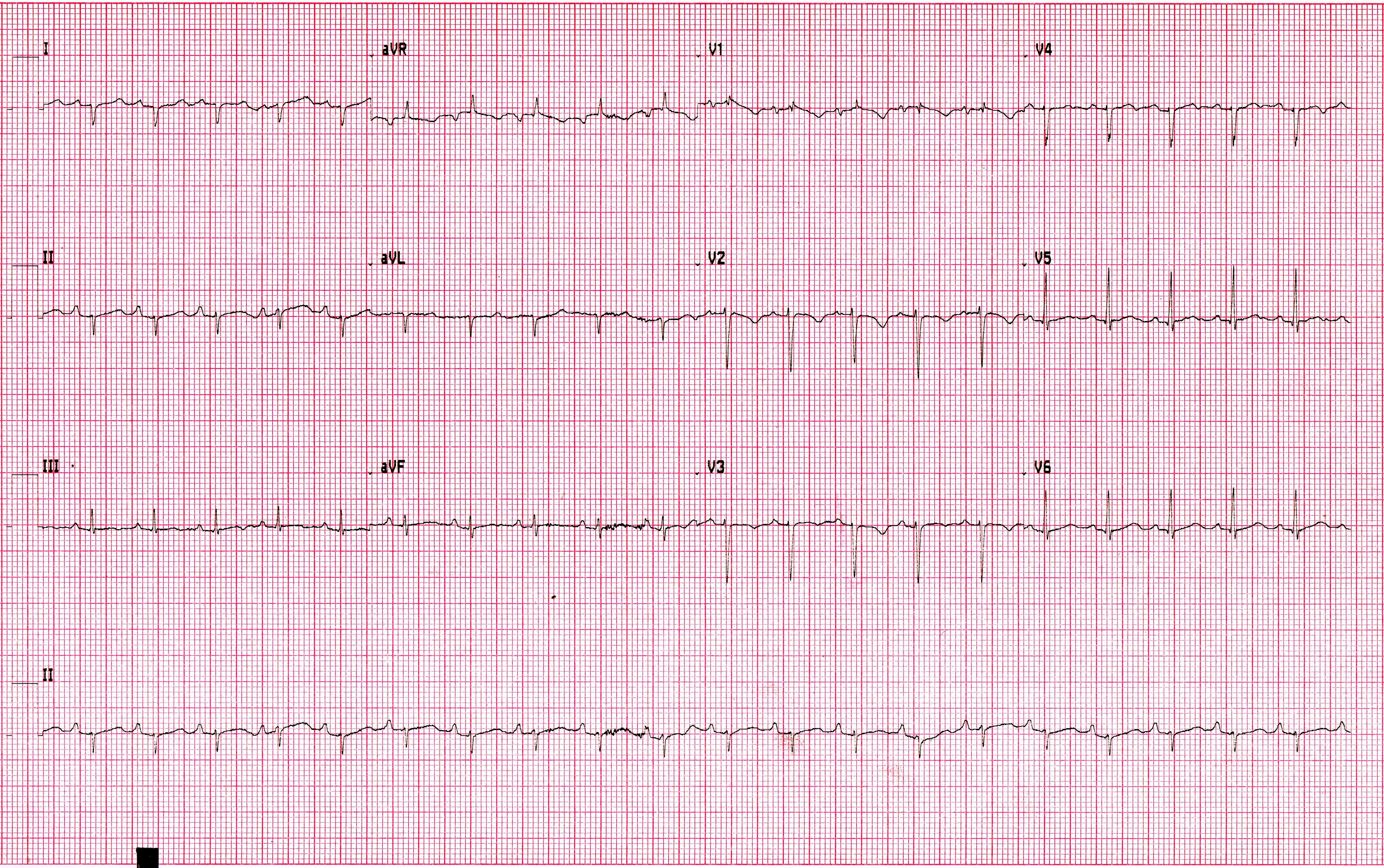

Supplement: Additional file 1: — The 12-lead electrocardiogram on presentation was notable for sinus tachycardia, low voltage (most prominent in limb leads) and electrical alternans (most prominent in precordial leads). [file 12969_2015_5_MOESM1_ESM.jpeg]
